# Visualization and Analysis of Eye Movement Data from Children with Typical and Atypical Development

**DOI:** 10.1007/s10803-013-1776-0

**Published:** 2013-02-05

**Authors:** Terje Falck-Ytter, Claes von Hofsten, Christopher Gillberg, Elisabeth Fernell

**Affiliations:** 1 Department of Women’s and Children’s Health, Center of Neurodevelopmental Disorders at Karolinska Institutet (KIND), Astrid Lindgren Children’s Hospital (Q2:07), 17176 Stockholm, Sweden; 2 Department of Psychology, Uppsala University, Box 1225, 751 42 Uppsala, Sweden; 3 Gillberg Neuropsychiatry Centre, Sahlgrenska Academy, Gothenburg University, Gothenburg, Sweden; 4 Autism Centre for Young Children, Habilitation and Health, Stockholm, Sweden

**Keywords:** Learning, Eye tracking, Scientific visualization, Bottom-up, Knowledge generation, Autism spectrum disorder, Diagnosis, Attention, Social dominance, Social hierarchies, Conflict, Goals

## Abstract

Looking at other children’s interactions provides rich learning opportunities for a small child. How children with autism look at other children is largely unknown. Using eye tracking, we studied gaze performance in children with autism and neurotypical comparison children while they were watching videos of semi-naturalistic social interactions between young children. Using a novel, bottom-up approach we identified event-related measures that distinguished between groups with high accuracy. The observed effects remained in a subset of the total sample matched on IQ, and were replicated across several different stimuli. The described method facilitates the detection of meaningful patterns in complex eye tracking data. Also, the approach significantly improves visualization, which will help investigators understand, illustrate, and generate new hypotheses.

## Introduction

Autism Spectrum Disorders (ASDs) are characterized by impairments within the domains of reciprocal verbal/non-verbal social interaction and by behaviors and interests that tend to be stereotyped, repetitive and ritualistic (Coleman and Gillberg 2012). The prevalence of ASD is assumed to be approximately 1 %. Most studies suggest a strong genetic basis, although an overly simplistic genetic view has been challenged (Hallmayer et al. 2011). ASDs are likely to be conditions associated with altered early developmental trajectories of brain structure and function (Elsabbagh et al. 2012; Gillberg 1999), and with considerable individual variability with regards to causes and course (Elsabbagh and Johnson 2010). Understanding attention in young children with ASD is critical in order to capture their view of the world—the information they attend to and the information they miss. Several recent studies using modern and non-invasive eye tracking technology have given new insights into the early phenotype of ASD (Chawarska et al. 2012; Klin et al. 2009; Fletcher-Watson et al. 2009; Falck-Ytter et al. 2012). However, the vast majority of eye tracking studies of early autism have not taken full advantage of both the spatial and the temporal resolution of the eye tracker. Indeed, attention in general, and in social contexts in particular, is a highly fluctuating phenomenon. For example, humans change their focus of foveal attention several times per second. Nevertheless, eye trackers are often used simply to measure whether the observer looks longer at one side of a computer screen than the other (e.g. Klin et al. 2009). These studies have revealed interesting results, but in order to understand the attentional processes, it is highly advantageous to develop techniques that allows for micro-level spatiotemporal analysis (Nakano et al. 2010; Klin et al. 2002). More specifically, it is critical to understand both *where* the participant looked, and *when* the participant looked there. The traditional AOI approach (looking time within a predefined area) largely misses the “when question”, and thus a key aspect of social attention. It is likely that many attentional differences between children with ASD and typical children are related to the exact timing of their eye movements, rather than to the distribution of gaze over long periods of time. At the same time, it is unlikely, that current theory can predict exact when and where these differences will occur. In this respect, bottom-up approaches can help us gaining new insight into typical and deviant neurodevelopmental processes.

**Fig. 1:**
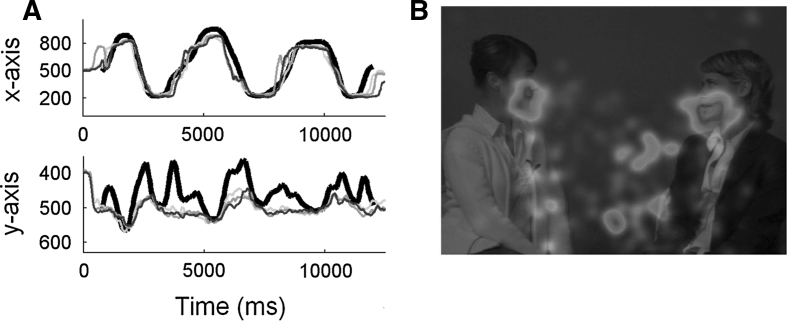
Traditional visualization of eye tracking data. **a** showing the (spatial) x- and the (spatial) y-dimension of eye tracking data in two different plots allows a completely lossless representation (time, x; time, y), but is difficult to make sense of intuitively. A central argument of this article is that it is rarely useful to know the absolute coordinates of the fixations. What is useful is to know their position relative to key elements of the stimulus. **b** Heat maps show unambiguously where on the screen the observers fixated, but contain no information about the timing of eye movements, and can be misleading if the stimulus is dynamic. Reproduced, with permission, from Falck-Ytter (2010) and von Hofsten et al. (2009)

Another challenge for eye tracking research is to develop efficient ways to visualize data. Good visualization is the key to understanding complex patterns of information (Card et al. 1999). However, current visualization tools for eye tracking are either highly complex (e.g. plotting x and y dimensions in separate plots; Fig. 1a), ignore the time dimension (e.g. heat maps; Fig. 1b), or fail to integrate information about the stimulus (Shic et al. 2012; Jones et al. 2012; Falck-Ytter 2010). Overcoming these limitations would be a substantial step forward, which would open up the scope for a wide range of applications. In addition, a highly meaningful, yet simple metric would increase chances that generic computer algorithms would detect the real effects present in the data (e.g. between group differences).

In essence, we believe that in order to be maximally informative to a human observer, an efficient format for spatial data from eye movement recordings needs to fulfill three criteria: it would have to (i) be representable in one dimension, allowing it to be plotted against time in 2D plot, (ii) relate to the content of the stimulus in a meaningful way, and (iii) be on a quantitative scale. In order to meet these criteria, it is necessary to reduce the two spatial dimensions contained in the original raw data stream to one dimension. One option would be to exclude either the x or y dimension, but such an approach would be clearly underspecified in many circumstances. In addition, knowing the absolute coordinates of gaze is rarely useful.

Here, we propose that a solution that meets all of the above criteria is to calculate the Euclidian distance from each individual’s gaze point to a key area in the scene, and do this for all time points in the data stream. We label this measure D2R (short for “distance to reference point”). This measure combines the two space dimensions into one dimension, but keeps the information that is needed to analyze the data with the naked eye in a two-dimensional space-by-time plot. While not suited for all studies or research questions, we would like to argue that D2R analysis is beneficial in many contexts.

**Fig. 2:**
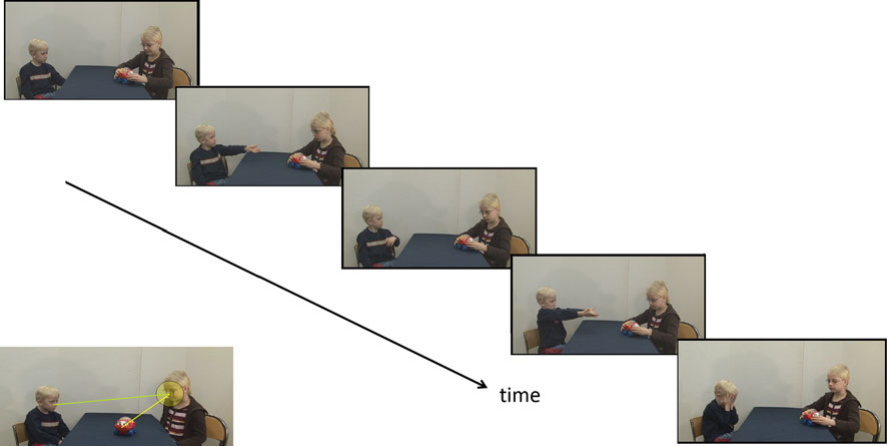
Stimuli. The *diagonal series* of pictures illustrates the course of events in the videos A–D, neither of which lasted more than 20 s. The *bottom left* picture illustrates the reference point (R; *center of yellow circle*), and a face AOI defined as distance to R (D2R) < 60 pixels. *Green* and *yellow lines* represent D2R from the boy and the toy, respectively (Color figure online)

Assume that the stimulus contains three spatially disparate elements (e.g., as in this study, face A, face B and an object; Fig. 2). This type of stimuli are frequently encountered in eye movement studies of children and infants. The distance from a fixation to, say, the center of face A, unambiguously specifies whether or not the observer looked at face A at all time points of the recording. Moreover, as long as the other elements (face B, the object) are not at an equal distance from face A, the same measure specifies (with high probability) whether the participant was looking at face B, the object or somewhere else. As this paper will illustrate, only one reference point (and thus, only one graph) is needed to achieve a rich and detailed representation of eye movements in this context. Also, if necessary, ambiguity can be easily resolved by adding a separate plot based on the distances to another reference point (e.g. face B).

For the purpose of illustrating this method, we showed semi-naturalistic videos to young children with ASD and to typically developing children. In the videos, two young children were engaged in toy directed activities and interacting using non-verbal gestures only (Fig. 2). Non-verbal communication impairments are part of the diagnostic definition of ASD (American Psychiatric Association 1994), and understanding of others’ actions has been suggested to be a key problem in children with ASD (von Hofsten et al. 2009). However, although we expected children with ASD to look at these events in a different way than other children, we had no a priori hypotheses about the exact nature of these differences. Indeed, we are not aware of any published eye tracking study that have looked at how young children with ASD look at other children interacting with each other—an ecologically valid stimulus with relevance for social function and learning (Bandura 1986). Thus, the current study is of both methodological and conceptual relevance.

## Methods

### Participants

Thirty-nine preschool children with a DSM-IV diagnosis of ASD [14 with Autistic Disorder (AD), 21 with Pervasive Developmental Disorder—Not Otherwise Specified [PDD-NOS] and four with Asperger Syndrome (AS)], and 28 typically developing (TD) children participated in the study (final samples after exclusion). Further details are given in Table 1. The children with ASD diagnoses were all recruited from a larger, community-based study of children in the county of Stockholm. Typically developing children were recruited from birth records in a university city in Sweden; respondents to our recruitment materials were predominantly Caucasian, middle-class families (see Table 1 for more information). For further information regarding recruitment and context, see Falck-Ytter et al. (2012).

**Table 1:** Study group characteristics

	ASD n = 39	TD n = 28
N girls	5 (13 %)	12 (43 %)
Age (years)	6.1/0.8 [4.0–7.3]	6.1/0.6 [4.3–7.3]
WPPSI-III total	81.0/13.4 [51–103]	115.1/13.5 [87–138]
WPPSI-III verb.	82.9/13.2 [53–114]	118.8/13.2 [93–143]
WPPSI-III perf.	89.7/16.4 [53–127]	111.8/15.6 [81–142]
VABS-II total	75.4/11.0	
ABC total	36.6/21.9	

Mean/SD [min–max]

In the ASD group, a diagnostic evaluation was made by a multidisciplinary team (consisting of neuropsychiatrist/paediatrician, neuropsychologist and speech language pathology therapist), and diagnoses according to the DSM-IV were made conjointly on the basis of all available information, which included clinical evaluation, DISCO-interview (Nygren et al. 2009), Autistic Behavior Checklist (ABC) (Krug et al. 1980), speech and language assessment, and assessment of cognitive (WPPSI-III; Wechsler 1967/2002) and adaptive functioning (Vineland Adaptive Behavior Scales—second edition; VABS-II; Sparrow et al. 2005).

Only children with a DSM-IV diagnosis of ASD according to this evaluation were included in the ASD group. From the larger study, only children who had valid cognitive test scores on the *Wechsler Preschool and Primary Scale of Intelligence* (Third Edition; WPPSI-III; Wechsler 1967/2002) were included in the current ASD group (the same test was administered to the TD group as well). TD participants were selected to match the ASD group in terms of chronological age (we also controlled for IQ in our analyses by comparing subsamples, see below). The TD group included slightly higher proportion of girls, facilitating analyses of gender differences in this group. We excluded children with known (uncorrected) visual or hearing impairments. One child with epilepsy was excluded from the TD group. No child in the TD group had a total IQ score below 70.

Parents provided written consent according to the guidelines specified by the Ethical Committee at Uppsala University and Karolinska Institute (the study was conducted in accordance with the standards specified in the 1964 Declaration of Helsinki).

### Stimuli

The stimuli were six short <20 s videos of semi-naturalistic social interactions between two young children (one slightly older than the other), shown in different orders (pseudo random) to each participant (Fig. 2). Each video was only shown once. When recording these videos, the actors were asked to perform certain actions according to a predefined script, but were asked to act as naturally as possible. The stimuli were accompanied by natural sound (the boy emphasized his request by vocalizing, and cried in videos where he did not receive the toy), but the actors did not speak to each other using words. The stimuli were mixed with regular attention grabber movies and other unrelated stimuli, as specified in Falck-Ytter et al. (2012).

### Procedure and Apparatus

The children were told that they were going to look at some short movies on the computer. In the TD group, WPPSI-III was administered after the eye tracking session. For ASD, clinical and cognitive assessments (see above) were conducted on a day other than the eye tracking session. As compensation for their participation, children/families received a small gift (value ~10 euro).

A corneal reflection technique (Tobii T120; Tobii Technology, Stockholm, Sweden) was used to record gaze of both eyes from the reflection of near-infrared light on the cornea and pupil at 60 Hz. An integrated 17′ TFT monitor was used to present the stimuli movies.

### Data Processing

Before exporting the eye tracking data to Matlab (Mathworks), we applied an I-VT filter (classifier: 30°/s; Velocity calculator window length: 20 ms) as provided in the software (Tobii Studio 3.03) provided by the eye tracker manufacturer. The output was based on the average of both eyes. No gap-filling or other noise reduction was used. To check whether the filter operated similarly in the two groups, we calculated the amount of time classified as fixations relative to the total time of raw data for each individual. On average for the ASD group, fixations were classified for 80.32 % (SD = 12.12 %) of the duration of the raw data. In the TD group, fixations were classified for 81.69 % (SD = 10.50) of the duration of the raw data, and there was no difference between the groups on this measure (t(65) = 482, ns; independent samples *t* test). The overall looking duration (after filtering) did not differ between the groups (mean/SD for ASD = 10.41/1.91 s; mean/SD for TD = 11.02/1.45; t(65) = 1.437, ns). The total number of fixations (per movie) was not significantly different between the groups (mean/SD for ASD = 36.05/12.10; mean/SD for TD = 45.00/22.36; ns, Mann–Whitney *U* Test). Similarly, the fixation rate (fixations per second) was equal across groups (mean/SD for ASD = 3.95/3.48 fixations/second; mean/SD for TD = 4.44/3.34; ns, Mann–Whitney *U* Test). In addition to these quantitative tests, visual inspection (using the gaze replay function provided by the Tobii Studio software; a dynamic visualization tool) supported the use of these settings. We discarded no data beyond what was excluded by the fixation filter.

**Fig. 3:**
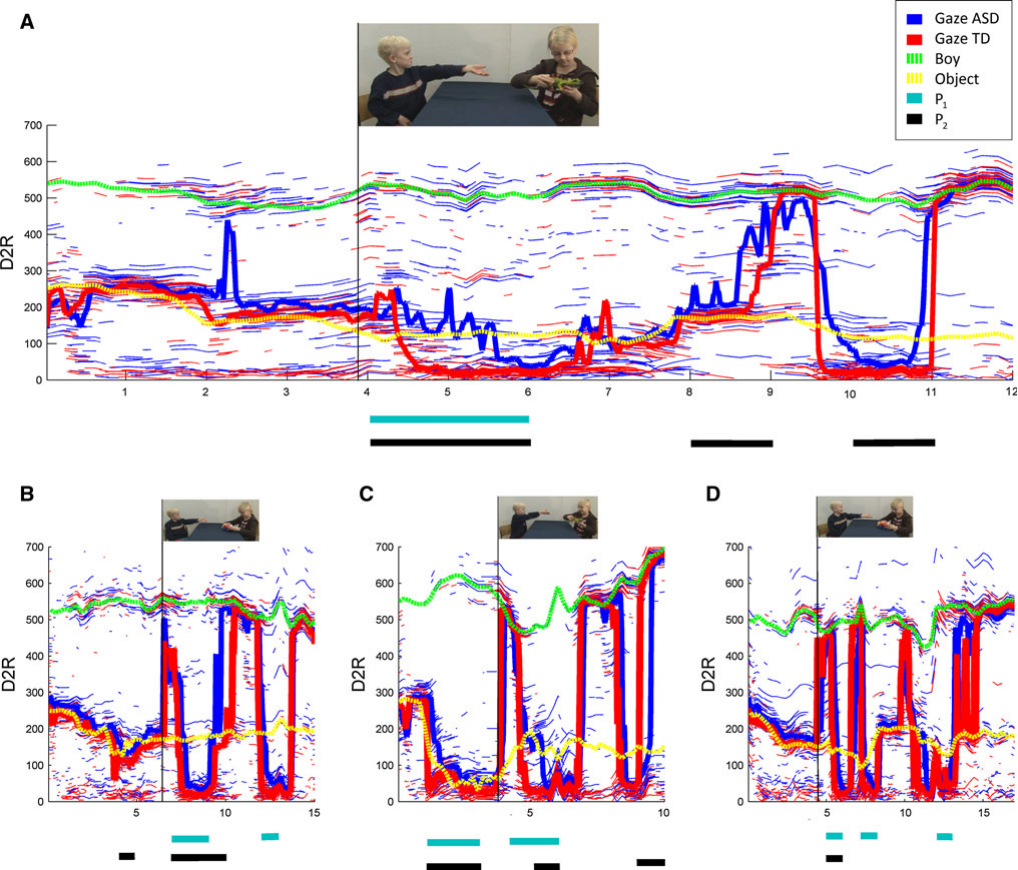
Visualizing three-dimensional eye tracking data in a two-dimensional graph. **a** Eye tracking data from children with TD and ASD during observation of video A (the seed for our analysis). The x-axis represents time (s), and the y-axis represents the Euclidian distance from the reference point (D2R, in pixels; R = the center of the girl’s face; D2R < 60 indicate fixations within her face). *Thin blue* and *red lines* represent D2R for individual fixations, while *thick blue* and *red lines* represent medians for each group. *Interrupted lines* represent D2R for two main objects in the stimulus: the boy (*green*) and the toy object (*yellow*; see Fig. 2 for supplementary illustration). Horizontal lines below graph index periods (1,000 ms bins) where the performance is significantly different between the two groups [alpha = .05; P_1_ (*cyan*) = *p* value for looking time within the girl’s face (D2R < 60); P_2_ (*black*) = *p* value for D2R]. Qualitative analysis of video content preceding the interval specified by both P_1_ and P_2_ (4–6 s) revealed an intuitive explanation for the effect: the onset of a requesting gesture (as indexed by a *vertical black line* and a static visual representation of the corresponding video content, *top*). In the TD group, this event was followed by fast fixations towards the girl’s face, while this response was delayed and weaker in the ASD group. **b**–**d** In three conceptually similar videos, the same pattern was found. Directly after the occurrence of a requesting gesture, both P_1_ and P_2_ flagged significant group differences (Color figure online)

We used in-house programs written in Matlab to (i) for each frame of the video extract the coordinates of key elements in the stimuli movies (the location of faces and toys), (ii) for each sample of the data stream, calculate the distance from the fixations to the reference point (D2R), (iii) visualize the result (Fig. 3), and (iv) perform bootstrapping (resampling, 10,000 iterations) in order to identify points in time (1,000 ms intervals) where the groups differed in overt attention. Remaining statistical computations were done in SPSS (SPSS Inc, Chicago, IL), with alpha-level .05 unless otherwise stated.

In this study, the reference point (R) was the center of the girl’s face (her nose; Fig. 2), unless otherwise specified. This choice was motivated by the fact that the two other key areas (the boy’s face, the toy object) were always at different distances from the girl’s face. Therefore, we expected this reference to be most informative. The performance of the in-house algorithm was validated by comparing its output to dynamic ‘gaze replays’ in Tobii Studio.

### Analytic Strategy

In order to test whether our bottom-up approach would produce valid results, we included several similar videos, analyzed them in a sequential manner, and tested (i) whether the identified instances in the first video would relate in a meaningful way to its content, and (ii) whether we could reproduce the results in similar movies (i.e. including qualitatively similar actions and reactions).

## Results

### Movie A

As a substrate for our analysis, we used a video in which the younger child’s request for the older child’s toy was ignored (Fig. 3a, see also Fig. 2). Our algorithm identified a period (4–6 s) where the two groups looking performance differed significantly both in terms of looking time within the girl’s face (D2R < 60) and in terms of D2R (Fig. 3a). During this period, children with typical development tended to focus on the girl’s face, while this tendency was weaker and delayed in children with ASD. Visual inspection of the video content preceding this interval revealed that the differential response occurred directly after the requesting actor’s imperative hand gesture. Thus, the algorithm identified an event that made sense intuitively: after watching a gestural request from one actor, the typical response is to look towards the other actor to see her reaction. The girl is in control over the resources (the toy) in this situation, and her face may help the observer predict the likely next course of events, a critical skill during social interaction (von Hofsten et al. 2009).

The algorithm also identified other intervals where there were group differences in the D2R measure (as indicated by black horizontal lines below the graph). During these periods, the groups did not differ in terms of their looking time within the girl’s face (D2R < 60), suggesting that these effects could relate to other parts of the scene. For example, between the eighth and the ninth second, the groups attended differently to the scene, but neither group tended to look at the girl’s face. To test this formally, we ran the analysis on video A again, now with the boy’s face as reference point. Given the spatial configuration of the video content, we expected this to reveal similar but fewer significant periods (because the girl’s face and the toy were frequently at the same distance to the boy’s face, and group differences with regards to these two elements therefore would not be detected). In accordance with this hypothesis, we identified only two periods (5–6 s; 8–9 s) that were flagged by both P_1_ and P_2_, and which coincided with the periods identified in Fig. 3a. This analysis confirmed that between the eighth and ninth second, the groups differed with regards to their looking time within the boy’s face. Again, this finding was strongly implicated already from in the first analysis (Fig. 3a). This illustrates the point that given appropriate stimuli and sensible choice of R, additional analyses may in fact not be needed.

### Movie B–D

Movie B, C, and D were conceptually similar to A. In movie B, the exact same instructions as in A were given to the actors (the only difference was the type of toy; dinosaur versus car), while in C (dinosaur) and D (car), the girl was instructed to refuse to give in a demonstrative manner (e.g. holding toy away from the requesting child). Thus, despite these differences in intensity and toy type, all videos followed the same basic social script. As can be seen in Fig. 3b–d, a similar pattern as in A was observed. That is, both P_1_ and P_2_ flagged significant differences directly after the onset of the requesting gesture of the youngest child. In addition, the algorithm identified several other discriminating periods of potential interest. However, for the sake of simplicity, and because these additional effects seemed to be more movie specific, we refrain from further discussion here.

### Classification Performance

**Fig. 4:**
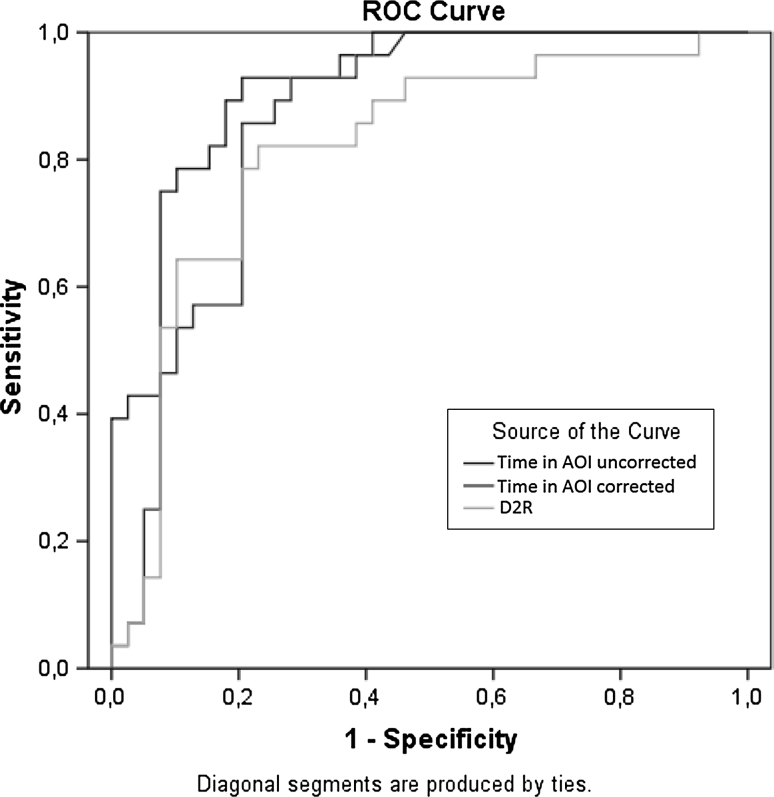
Classification performance of three eye tracking measures. The ROC curves plot the relationship between the true positive rate and false positive rate across the full range of possible thresholds available. There was no significant difference in classification performance between the traditional AOI-based measures and the D2R measure

Our results suggest that videos A–D elicit predictable differences in looking performance between children with and without ASD. In all four videos, the same pattern emerged. Given the similarity in both video content and results, we combined (averaged) the data from the most discriminative 1-s bin following the imperative gesture, from each of the four videos. This was done separately for spatial and duration data. We applied a Receiver Operating Characteristics (ROC) curve analysis, which illustrates classification accuracy by plotting the sensitivity and specificity of a test across the whole range of possible performance criteria. For the time constrained AOI measure [looking time within 60 pixels from reference point (center of the girls face)], Area Under the Curve (AUC) was excellent (.916; Fig. 4). However, since this result could reflect overall shorter looking time (across the whole display) for ASD during the test interval, we also calculated looking time within the AOI relative to the whole screen for each movie (during the 1-s time bin), and calculated the mean of these four values. For this corrected AOI measure, the AUC remained high (.855). Most importantly for the purposes of this article, for the D2R measure, AUC was high (.815), and not significantly different from any of the two time based measures (z < 1.49, ns). Thus, the D2R measure performed equally well as the traditional AOI measures.

### Controlling for Cognitive Ability

The groups differed in terms of IQ, and in order to rule out cognitive ability as a confounder, we selected two subsamples, attempting to reach similar levels of IQ across the two groups. We used total IQ scores as the matching variable (Rice et al. 2012) (Table 2), and compared the groups on the average score from movie A–D (as in the ROC analysis). Chronological age was unrelated to the looking measures in both groups, and thus, we did not take chronological age into account when matching. We found that the corrected AOI measure (see above) differentiated between the groups (t(14) = −3.440, *p* = .008, independent samples *t* test, one tailed; the uncorrected AOI measure showed an even stronger effect). Similarly, the D2R measure differed between the two IQ matched groups (t(14) = 2.135, *p* = .026, one-tailed). Thus, the different gaze patterns do not appear to be explained by IQ.

**Table 2:** Study group characteristics, IQ-matched subsamples (mean/SDs)

	ASD (n = 8)	TD (n = 8)	Pairwise comparison (*p* value)^a^
N girls	1	4	
Age (years)	5.8/0.8	5.8/0.8	n.s.
WPPSI-III total	96.1/9.2	97.9/7.4	n.s.
WPPSI-III verb.	96.5/10.8	106.1/9.7	n.s.
WPPSI-III perf.	108.0/11.2	93.5/7.8	.011
VABS-II total	80.5/16.0		
ABC total	36.6/19.3		

^a^Independent samples *t* test

### Correlations with Test Scores

In each group, we correlated the performance on the eye tracking task (averaged across video A–D, as in the ROC analysis above) with the instruments used for diagnostic evaluation and/or participant characterization (WPPSI-III, ABC, VABS-II). In the ASD group, the VABS-II communication scale and the WPPSI-III verbal scale correlated negatively with the D2R measure (r = −.321, *p* = .46 and r = −.400, p = .012 respectively, Pearson correlation). Thus, better verbal and communicative ability predicted fixations closer to the center of the girl’s face during the time window of interest. No other correlations were found. In the TD group (who had WPPSI-III data, but not ABC and VABS-II data), a positive correlation was found between the corrected AOI looking time measure and WPSSI-III performance (r = .514, *p* .005). No other correlations were found.

### Gender Differences

The ASD sample included only five girls, and therefore no attempt to analyze gender differences in this group was made. In the TD group, we found no gender differences, neither in terms of the corrected AOI measure (t(26) = 1.485, ns, independent samples *t* test) nor in terms of D2R (t(26) = .905, ns; analyses based on average scores from movie A–D, as in the ROC analysis above). Thus, the slightly higher proportion of girls in the TD sample is unlikely to explain the differences related to diagnostic status. To investigate this more directly, we excluded all girls from both samples and compared the two boy groups (TD vs ASD) in terms of the corrected AOI (looking time) measure and the D2R measure. Both tests were highly significant (t(48) = 3.724, *p* = .001, and (t(48) = 3.385, *p* = .001, independent samples *t* test).

### Generalizability with Regards to Interaction Type

In order to test whether the observed pattern (Fig. 3a–d) might generalize to friendly interaction, we also applied the analysis to two videos showing interactions where the request was followed by receiving the toy from the girl. As before, each video was only shown once. The results were mixed. For one video the above (A–D) pattern was repeated (both P_1_ and P_2_ flagged a significant group difference directly after the occurrence of the gesture). In other words, during the first seconds following the boy’s gesture, the children with TD looked more consistently at the girl’s face than the children with ASD. However, in the second video, no such group difference was found.

## Discussion

We have described a novel method that allows both for efficient visualization of eye tracking data and for automatic detection of events that elicit different responses in children with typical development and children with a neurodevelopmental disorder (ASD). This method established that after having observed a non-verbal request for a toy, typical children tend to immediately turn their attention to the face of the child holding the toy in question (the girl). This performance is probably highly adaptive, because the girl, at this very point in time, is the one in power to determine what is going to happen next. Children with ASD showed a much weaker tendency to look towards the girl after having seen the boy’s gesture, suggesting that when observing dynamic, non-verbal interaction between other children, these children may fail to follow the course of events as efficiently as their typically developing peers. Supplementary analyses suggested that these group differences could not be explained by differences in intelligence or gender. As can be seen in Fig. 3, rather than being attracted by a single feature, we observed substantial individual differences in ASD during the critical time windows (see blue lines, e.g. between the fourth and sixth second in Fig. 3a). Failing to look at the ‘right’ places during critical phases of a social interaction may have negative consequences for children’s opportunities to learn from what they are seeing, their ability to anticipate what is going to happen next, and—ultimately—their ability to interact with other people (Bandura 1986; von Hofsten et al. 2009).

In addition to being able to classify TD vs ASD as well as traditional AOI looking time measures, our new measure (D2R) has the important advantage that it can be plotted against time to give a rich, yet simple, visual presentation of both spatial and temporal aspects of gaze performance. As Fig. 3 illustrates, when plotted against time, the D2R measure captures both where the children looked, and when they looked there. It has the ability to detect instances of high cohesion, while at the same time linking these events to the content of the stimulus. Thus, the D2R measure has characteristics optimal both for visualization of dynamic events, and for related statistical analyses.

To be able to visualize, in a direct and intuitive way, the data to be used for statistical analysis is clearly beneficial. The traditional AOI approach lacks this property. The most widely used AOI measure is relative looking time in an AOI. To calculate this, you need to sum up the data within an area, and divide this sum with the total looking time across the whole display (Falck-Ytter et al. 2010). Although spatial data can be effectively visualized in heat maps (Fig. 1b), the temporal dimension is ignored in such displays. In the current study, the temporal aspect was found to be critical. Visualization is useful for detection of unexpected patterns, finding alternative explanations and generating new hypotheses (Fox and Hendler 2011). Visualization also has an important function during initial data quality checks (Yu et al. 2012), and in scientific communication.

Given the classification performance observed in this study, the present results are in line with the idea that eye tracking methodology could be used as a complement to traditional diagnostic instruments in the future (Pierce et al. 2011). The fact that eye tracking can be used to assess social processing skills in just a few minutes adds to this argument. Further studies, in particular studies including other neurodevelopmental disorders than ASD are needed to evaluate this hypothesis directly. At present, we have no evidence that the pattern observed in this study is specific to ASD.

The strength of naturalistic studies is their ability to generate new ideas and their ecological validity (Yu et al. 2012; Noris et al. 2012). These new ideas can be tested in further experiments. Our study was semi-naturalistic as it included un-edited scenes of two children interacting with each other. Generalizability to real life is still limited, though, due to the use of video and to the fact that the actors were instructed to perform certain actions. Interestingly, the same pattern of results was stable within the antagonistic videos (A–D), but generalized to only one of the non-antagonistic videos. Although strong hypotheses about the observed pattern are premature, one may speculate that subtle cues of antagonism prior to the gesture moderate the tendency to look towards the girl in the present context. Interpreted from this perspective, the findings motivate further study of how children with ASD process cues of social power and social dominance in the context of conflicting goals. Alternatively, it is possible that the tendency to look to the girls face could be a more general response, but that irrelevant cues/events happened to distract the TD group in one of the non-antagonistic movies included in this study. It is worth noting that in the current within-subject design, the children can build up expectation about what is going to happen based on previous movies. Thus, the group differences could also be related to memory. Finally, the current study investigated how children look at two other children in a context where they were not themselves active in the interaction. To map out how children with ASD look in a real social interaction has not been studied in much (but see Doherty-Sneddon et al. 2012). Current efforts are underway in our lab to evaluate the value of D2R analysis in other contexts, including in live-eye tracking experiments.

Our results suggested that in the children with ASD, better performance on the eye tracking task (i.e. behaving more like the TD group) was associated with having higher verbal IQ and having better adaptive communication skills. This fits well with the idea that how you look in a social context relates to your ability to communicate. To more precisely specify the nature of this association would be important from a developmental perspective. In the TD group, non-verbal IQ predicted more looking towards the girl’s face after seeing the boy’s gesture. This points to the possibility that the same behavior may be linked to partially different processes in the two child groups.

Although we have used the term data-driven to describe the current approach, we should emphasize that it requires some ideas about the effects one is looking for. Specifically, one needs to determine the reference points to be feed into the D2R analysis. This step is important and will be directly reflected in the output, and may be more difficult than in the current study depending on the content of the stimuli. One single D2R analysis can handle complexity as long as distances from the reference point to the other objects are not identical. Frequently, stimuli are made with a specific analysis in mind, allowing the researcher to maximize the power of the chosen analytic procedure. We did not select the hands as reference points. The reason for this was that often the hands were spatially indistinguishable from the toy object. It is not unlikely that many of the fixations in the 100-400 pixel range in Fig. 3a are in fact directed towards the hands of the actors.

The D2R measure can be expressed on a ratio scale for all time points of the data stream. Thus, in principle, the chances to detect statistical significant differences do not change as a function of the length of the time-interval of interest. In contrast, looking time within an AOI is reduced to a dichotomous measure if the test interval is minimized (1 sample), with associated loss of statistical power. Another technical advantage of D2R is its robustness to temporal noise (data loss). In traditional AOI analysis, this can be controlled for (and frequently is, as in this study) by calculating relative looking time in an AOI, but no such extra step is required for D2R analysis. D2R analysis provides some robustness to spatial noise as well. Provided that the noise is evenly scattered around the true fixation point and the subject is fixating somewhere else than on the reference point itself, the D2R measure will be accurate when averaged over time. In contrast, even evenly scattered noise will tend to bias measures of looking time within AOI (Wass et al. in press).

We deliberately chose a liberal alpha level for statistical tests (.05). We believe that during initial phases of hypothesis generation, this is reasonable. What is important, however, is that once a hypothesis has been formulated, it has to be tested on a new dataset (as we did in movie B–D).

It is important to stress that we would not have obtained the present results if we ran the analysis on only one spatial dimension of the data (e.g. the x-dimension). For example, as can be seen in Fig. 3a, only focusing on the x-dimension would mean missing that the two groups differed during the 4–6 s interval (because the ASD group tended to look towards the toy and the TD group tended to look at the girl’s face and that these two objects had similar x coordinates; see static stimulus representation in Fig. 3a, top). Although it is possible to visualize both the x, y and time dimensions in 3D plots, these plots tend to be very complex and thus hard to analyze with the naked eye. Such visualization gets even more complex if one tries to incorporate information about the dynamic stimulus content, information that is naturally embedded in the much more simple D2R analysis.

ASDs are characterized by variant developmental trajectories early in life, variants that have cascading consequences for socio-cognitive development and for social functioning later on. Eye tracking research on young children with ASD is one way to understand these complex, and subtle, processes. We have presented a novel measure, labeled D2R, that can be used both for mathematical and visual representations of eye tracking data. Using this method, we found that young children with ASD look at other children differently, and provided direct clues regarding the specific information they may fail to attend to. Thus, our study has implications for theories of ASD, for methodological development, as well as for clinical applications of novel methods in the field of early autism.

## Abbreviations

ASD: Autism spectrum disorder
TD: Typically developing
AOI: Area of interest
D2R: Distance to reference point
